# Serum Alpha-Fetoprotein as a Predictor of Liver Fibrosis in HBeAg-Positive Chronic Hepatitis B Patients

**DOI:** 10.3390/medicina59050923

**Published:** 2023-05-11

**Authors:** Kai Yang, Ying Pan, Liwei Liu, Beibei Sun, Wei Shi

**Affiliations:** 1 Department of Medical Technology, Anhui Medical College, Hefei 230601, China; 2 Department of Clinical Laboratory, The Second Affiliated Hospital of Anhui Medical University, Hefei 230601, China

**Keywords:** alpha-fetoprotein, biomarker, liver fibrosis, chronic hepatitis B

## Abstract

*Background and Objectives*: Non-invasive methods for evaluating liver fibrosis have been a crucial focus of clinical research. The aim of the current study is to assess the accuracy of serum alpha-fetoprotein (AFP) in determining the stage of liver fibrosis in patients with chronic hepatitis B (CHB) who are positive for HBeAg. *Materials and Methods*: The current study included a total of 276 HBeAg-positive CHB patients who underwent liver biopsy. The levels of serum AFP were measured in these patients using electrochemiluminescence immunoassays. The correlations between serum AFP levels and other laboratory parameters were analyzed using Spearman’s correlation analysis. Binary logistic regression analysis was performed to determine the independent associations between serum AFP levels and liver fibrosis. The diagnostic performance of serum AFP and other non-invasive markers was evaluated using receiver operating characteristic (ROC) curves. *Results*: A total of 59 (21.4%) patients were found to have elevated levels of serum AFP (>7 ng/mL). These patients displayed a significantly higher proportion of both advanced fibrosis and cirrhosis compared to those with normal serum AFP levels (0–7 ng/mL). The level of serum AFP was positively associated with levels of serum globulin (GLB), alanine aminotransferase (ALT), aspartate aminotransferase (AST), and total bilirubin (TBIL), as well as the AST-to-platelet ratio (APRI), fibrosis-4 (FIB-4), and Scheuer’s classification, and negatively correlated with platelet (PLT) counts. Furthermore, serum AFP was found to be independently associated with significant fibrosis, advanced fibrosis, and cirrhosis. The results of the ROC analysis showed that serum AFP was an effective predictor of significant fibrosis, advanced fibrosis, and cirrhosis, with an area under the receiver operating characteristic curve (AUROC) of 0.773 (95% CI: 0.721–0.821), 0.889 (95% CI: 0.847–0.923), and 0.925 (95% CI: 0.887–0.953), respectively. These values are higher than those of the APRI and FIB-4. *Conclusions*: Serum AFP could serve as a valuable supplemental biomarker for determining the severity of liver fibrosis in HBeAg-positive patients with chronic hepatitis B.

## 1. Introduction

The Hepatitis B virus (HBV) is a human pathogen that causes chronic hepatitis B (CHB), and it is estimated to affect over 350 million people worldwide, with approximately 30 million of those cases located in China [1]. If left untreated, CHB progresses to liver cirrhosis, liver failure, and ultimately to hepatocellular carcinoma (HCC) [2]. The intermediate stage between CHB and cirrhosis is liver fibrosis, which can be divided into five stages using Scheuer’s classification [3]. In clinical practice, determining the stage of liver fibrosis is crucial in managing CHB [4]. Liver biopsy is currently considered the gold standard for assessing liver fibrosis stage, but it is invasive, requires specialized equipment and technical expertise, and carries potential life-threatening complications, limiting its clinical use [5]. Non-invasive methods, such as transient elastography (TE), have emerged as promising alternatives for staging liver fibrosis. TE is an ultrasonography-based method that is safe, but its accuracy may be affected by factors such as cholestasis, body mass index (BMI), and hepatic inflammatory activity [6]. Additionally, non-invasive fibrosis scores, such as the AST-to-platelet ratio (APRI) and fibrosis-4 (FIB-4), which are based on laboratory parameters, have also been proposed for use in regions with limited healthcare resources [7]. While these scores offer the advantage of cost reduction, their diagnostic performance falls short of meeting clinical requirements due to their moderate accuracy, typically characterized by an area under the receiver operating characteristic curve (AUC) ranging from 0.7 to 0.8 [8].

Alpha-fetoprotein (AFP) is a glycoprotein primarily produced by the yolk sac and fetal liver [9]. In newborns, its serum level is high, but in healthy adults, it decreases to below 7 ng/mL. An abnormal increase in the serum AFP level in adults is often considered an indicator of liver disease, particularly HCC [10]. However, serum AFP has limited utility in HCC diagnosis as it can also increase in other non-HCC diseases, such as autoimmune hepatitis (AIH) and acute liver failure (ALF) [11]. A study from China found that 27.2% of CHB patients had an AFP level above the normal range [12]. Serum AFP has been shown to effectively predict liver histology in HBeAg-negative CHB patients [13]. A Japanese study also found that low baseline levels of serum AFP are assocJiated with fibrosis regression in hepatitis C virus (HCV) infected patients who achieved sustained virological response (SVR) [14]. In this study, we investigated the diagnostic value of serum AFP for liver fibrosis in HBeAg-positive CHB patients, as most CHB patients in China are HBeAg-positive.

## 2. Materials and Methods

### 2.1. Study Population

The current study included 276 HBeAg-positive CHB patients who underwent a liver biopsy to assess the stage of liver fibrosis between 2015 and 2021 in the Department of Infectious Diseases at the Second Hospital of Anhui Medical University. The diagnosis of CHB was based on the “Guidelines for the prevention and treatment of chronic hepatitis B” issued by the Chinese Medical Association. The study participants were eligible if they were over 16 years old, had positive HBsAg, HBeAg, and HBV DNA for at least 6 months, and had no history of antiviral treatment. The exclusion criteria for patients included co-infection with HCV or human immunodeficiency virus (HIV), the coexistence of HCC, and decompensated liver cirrhosis. The study was conducted in accordance with the Helsinki Declaration of 1983 and was approved by the Ethics Boards of Anhui Medical College and the Second Hospital of Anhui Medical University. All participants provided informed consent.

### 2.2. Liver Biopsy

All recruited patients underwent ultrasound-guided percutaneous liver biopsy within 48 h of admission. The biopsy specimens were immediately fixed in 10% neutral formalin after collection and then embedded in paraffin. The samples were processed with hematoxylin-eosin and Masson trichrome stains. Two experienced liver pathologists, who were blinded to the results, utilized the Scheuer classification to evaluate the stage of liver fibrosis (ranging from S0 to S4) in a cohort of 276 patients. Among the patients, the two pathologists had concordant results in determining the stage of liver fibrosis in 251 cases. In the remaining 25 patients, a third pathologist was consulted to provide assistance and arrive at a final conclusion regarding the stage of liver fibrosis. In accordance with the classification method proposed by Wang et al. [5], significant fibrosis was defined as the range of S2 to S4. Advanced fibrosis was defined as the range of S3 to S4, while cirrhosis was defined specifically as S4.2.3. Laboratory tests.

Blood samples were collected from patients on the day prior to the liver biopsy and were analyzed immediately. Biochemical parameters, such as total protein (TP), albumin (ALB), globulin (GLB), alanine aminotransferase (ALT), aspartate aminotransferase (AST), and total bilirubin (TBIL), were measured using the Beckmann Coulter AU5800 biochemical analyzer (Beckman Coulter, Brea, CA, USA). PLT counts were determined using the Sysmex XE2100 automated hematology analyzer (Sysmex Corporation, Kobe, Japan). The serum AFP levels were determined using the Cobas E601 chemiluminescence analyzer (Roche Diagnostics, Basel, Switzerland). The serum HBV DNA levels were quantified using the Strata Gene MX3000P Detection System (Stratagene, La Jolla, CA, USA), while serum HBsAg and HBeAg levels were measured using the ARCHITECT i2000SR system (Abbott Laboratories, Chicago, IL, USA).

### 2.3. Statistical Analysis

The statistical analysis was carried out using SPSS software (version 19.0, SPSS Inc., Chicago, IL, USA) or MedCalc statistical software (version 20.118, MedCalc Software Ltd., Ostend, Belgium). Categorical variables were presented as the number (*n*) and percentage (%), while continuous variables were shown as mean ± standard deviation (SD) or median with interquartile range (IQR), as appropriate. The chi-square test was used to compare categorical variables, while the Student’s *t*-test or Mann–Whitney U test was used for continuous variables. The correlation between different variables was analyzed using Spearman’s rank correlation coefficient. Univariate and multivariate logistic regression analysis was performed to determine the independent factors associated with liver fibrosis. The diagnostic accuracy of AFP was evaluated using a receiver operating characteristic (ROC) curve. A *p*-value of less than 0.05 was considered statistically significant for all tests.

## 3. Results

### 3.1. Study Population

The demographic and clinical data for the patients are summarized in Table 1. The median age was 33.00 years, and males comprised 81.2% of the patients. Liver fibrosis stage, as determined by liver biopsy, was classified as follows: S0 (*n* = 7, 2.54%), S1 (*n* = 145, 52.54%), S2 (*n* = 82, 29.71%), S3 (*n* = 29, 10.51%), and S4 (*n* = 13, 4.71%). The median values of APRI and FIB-4 were 0.68 and 1.00, respectively. Overall, 217 (78.6%) patients had normal serum AFP levels (0–7 ng/mL), and 59 (21.4%) patients had elevated serum AFP levels (>7 ng/mL). Patients with high serum AFP levels had lower ALB (*p* = 0.004), higher GLB (*p* = 0.002), higher ALT (*p* < 0.001), higher AST (*p* < 0.001), higher TBIL (*p* < 0.001), higher APRI scores (*p* < 0.001), higher FIB-4 scores (*p* < 0.001), and a higher proportion of advanced fibrosis (≥S3) (*p* < 0.001) and cirrhosis (≥S4) (*p* < 0.001) compared to patients with normal serum AFP levels.

### 3.2. Association between Serum AFP Levels and Laboratory Findings

**Figure 1:**
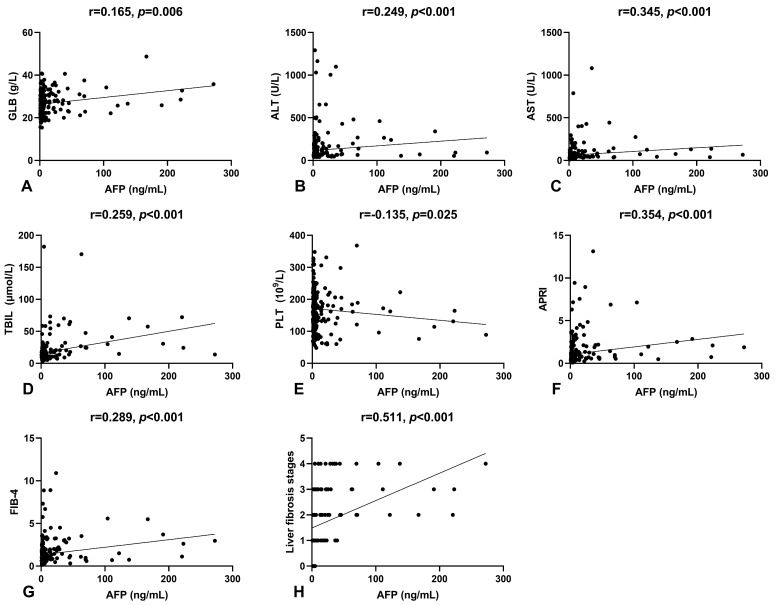
The correlation of serum AFP levels with other indicators. (**A**) The correlation between serum AFP and GLB levels. (**B**) The correlation between serum AFP and ALT levels. (**C**) The correlation between serum AFP and AST levels. (**D**) The correlation between serum AFP and TBIL levels. (**E**) The correlation between serum AFP levels and platelet counts. (**F**) The correlation between serum AFP levels and APRI. (**G**) The correlation between serum AFP levels and FIB-4. (**H**) The correlation between serum AFP levels and liver fibrosis stages.

Spearman’s correlation analysis was conducted to examine the relationship between serum AFP levels and other laboratory parameters in patients. Results indicated a positive correlation between serum AFP levels and GLB (r = 0.165, *p* = 0.006), ALT (r = 0.249, *p* < 0.001), AST (r = 0.345, *p* < 0.001), and TBIL (r = 0.259, *p* < 0.001), and a negative correlation with PLT (r = −0.135, *p* = 0.025), as shown in Figure 1A–E. To further investigate the clinical relevance of serum AFP levels in HBeAg-positive CHB patients, we analyzed its correlation with non-invasive fibrosis scores and Scheuer’s classification, as shown in Figure 1F–H. Serum AFP levels significantly correlated with APRI (r = 0.354, *p* < 0.001) and FIB-4 (r = 0.289, *p* < 0.001). Notably, there was a moderate positive correlation between serum AFP levels and Scheuer’s classification (r = 0.511, *p* < 0.001), suggesting that serum AFP could be a potential biomarker for liver fibrosis.

### 3.3. Serum AFP Level Is Independently Associated with Liver Fibrosis

We conducted univariate and multivariate logistic regression analyses to identify independent factors associated with liver fibrosis, as presented in Table 2. The univariate analysis results showed that age (*p* = 0.002), ALT (*p* = 0.021), AST (*p* = 0.003), TBIL (*p* < 0.001), PLT (*p* = 0.001), and AFP (*p* < 0.001) were significantly associated with significant fibrosis. However, the multivariate analysis revealed that only PLT (*p* = 0.018) and AFP (*p* = 0.001) remained independent factors for significant fibrosis. Additionally, further analysis demonstrated that AFP was also an independent factor for advanced fibrosis (*p* = 0.002) and cirrhosis (*p* = 0.010).

### 3.4. Diagnostic Performance of Serum AFP in Predicting Liver Fibrosis

**Figure 2:**
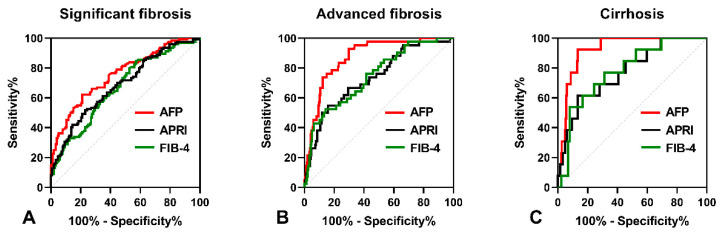
The ROC curves comparing the performance of serum AFP levels, APRI and FIB-4. (**A**) The ROC curves for diagnosing significant fibrosis. (**B**) The ROC curves for diagnosing advanced fibrosis. (**C**) The ROC curves for diagnosing cirrhosis.

As shown in Figure 2A, the AUROC of serum AFP was 0.773 (95% CI: 0.721–0.821), which was higher than that of APRI (AUROC = 0.686, 95% CI: 0.629–0.739, *p* = 0.012) and FIB-4 (AUROC = 0.705, 95% CI: 0.648–0.757, *p* = 0.049) in predicting significant fibrosis. The optimal cut-off point for serum AFP was 3.6 ng/mL, with a sensitivity of 65.19% and a specificity of 78.95%. In predicting advanced fibrosis, the AUROC of serum AFP (0.889, 95% CI: 0.847–0.923) was higher than that of APRI (0.757, 95% CI: 0.703–0.806, *p* < 0.001) and FIB-4 (0.772, 95% CI: 0.719–0.819, *p* = 0.003), as shown Figure 2B. The optimal cut-off point for serum AFP was 6.22 ng/mL, with a sensitivity of 81.13% and a specificity of 85.04%. Furthermore, the AUROC of serum AFP in predicting cirrhosis (0.925, 95% CI: 0.887–0.953) was higher than that of APRI (0.773, 95% CI: 0.719–0.821, *p* = 0.046) and FIB-4 (0.781, 95% CI: 0.727–0.828, *p* = 0.028), as shown in Figure 2C. The optimal cut-off point for serum AFP was 9.88 ng/mL, with a sensitivity of 92.31% and a specificity of 86.69%.

## 4. Discussion

In this study, we examined the efficacy of serum AFP in predicting liver fibrosis in HBeAg-positive CHB patients. Our results revealed that elevated serum AFP levels were not only associated with liver injury but also served as an independent predictor of liver fibrosis. Moreover, serum AFP demonstrated superior diagnostic accuracy over APRI and FIB-4 in forecasting significant fibrosis, advanced fibrosis, and cirrhosis in HBeAg-positive CHB patients.

Liver fibrosis is a well-known pathological response to various types of chronic liver injury, which significantly impairs liver function [15]. In chronic HBV infection, incomplete activation of the hepatic immune system fails to eliminate HBV from hepatocytes, leading to persistent liver damage. This process is often accompanied by hepatocyte necrosis, post-necrotic hepatocellular regeneration, and deposition of extracellular matrix [16], resulting in a substantial proportion of CHB patients with necrotic inflammation, fibrosis, and even cirrhosis on liver biopsy. In recent decades, there have been numerous reports on the diagnostic value of serum AFP for liver fibrosis in patients with HCV infection. Several studies have demonstrated that elevated serum AFP levels are highly specific for the diagnosis of advanced fibrosis and cirrhosis in patients with chronic Hepatitis C (CHC) [17,18]. However, it is less clear whether serum AFP has similar diagnostic utility for liver fibrosis in CHB patients as compared to CHC patients. Liu et al. demonstrated that serum AFP levels are increased in CHB patients and are significantly associated with the liver fibrosis stage [19]. In HBeAg-negative CHB patients, Lee et al. reported that serum AFP independently correlated with the liver fibrosis stage, suggesting a potential role for serum AFP as a non-invasive diagnostic approach for liver fibrosis [13]. Nonetheless, further studies are necessary to investigate whether serum AFP has similar diagnostic utility in evaluating HBeAg-positive CHB patients with varying degrees of liver fibrosis.

In this study, 24.1% of patients with HBeAg-positive CHB showed elevated serum AFP levels beyond the upper limit of normal. These patients had a poorer liver function and a higher prevalence of severe fibrosis/cirrhosis compared to those with normal AFP levels. Our findings showed positive associations between serum AFP levels and liver function parameters such as GLB, ALT, AST, and TBIL, which are components of non-invasive liver fibrosis scoring systems such as GP, FIB-4, APRI, and ALBI scores [20,21]. Additionally, serum AFP levels were inversely correlated with PLT count. We found significant positive associations between serum AFP levels and both APRI and FIB-4 scores, as expected. Our data suggest that serum AFP levels increase with the severity of liver lesions, supported by the finding that serum AFP levels were elevated in patients with higher fibrosis stages.

Although the exact mechanism underlying the positive association between serum AFP levels and the degree of liver fibrosis remains to be studied, it may be related to the crosstalk between hepatocytes and activated hepatic stellate cells (HSCs), which can generate a pro-fibrotic liver microenvironment during HBV infection. It is well-known that HSC activation is the critical event underlying liver fibrogenesis, and HBV can induce HSC activation through multiple mechanisms [22,23]. In a study of liver fibrotic murine, it was shown that activated HSCs could promote AFP expression in adjacent hepatocytes [24]. Moreover, AFP was recently reported to promote the expression of epithelial cell adhesion molecule (EpCAM), which plays an important role in the activation and proliferation of HSCs [25]. These findings support our results regarding the positive relationship between serum AFP levels and the degree of liver fibrosis. Further analysis by logistic regression showed that serum AFP was independently associated with significant fibrosis, advanced fibrosis, and cirrhosis. These findings strengthen the possibility that serum AFP is a potential biomarker for liver fibrosis in HBeAg-positive CHB patients.

APRI and FIB-4 are non-invasive indices recommended by the World Health Organization (WHO) for evaluating the liver fibrosis stage in CHB patients [26]. In this study, we compared the diagnostic performances of serum AFP with APRI and FIB-4 in predicting significant fibrosis, advanced fibrosis, and cirrhosis in HBeAg-positive CHB patients. The results showed that the AUROC of serum AFP was higher than that of FIB-4 and APRI for predicting significant fibrosis. For predicting advanced fibrosis and cirrhosis, serum AFP had a significantly higher AUROC compared to APRI and FIB-4. Notably, serum AFP exhibited the highest AUROC in diagnosing cirrhosis compared to significant fibrosis and advanced fibrosis. Overall, these findings suggest that serum AFP is a superior predictor of fibrosis stage in HBeAg-positive CHB patients compared to APRI and FIB-4.

Our study had some limitations. Firstly, it was a single-center retrospective study, and selection bias was inevitable. Secondly, serum AFP levels were not dynamically detected in this study. Further studies are needed to investigate whether serum AFP can predict fibrosis regression during antiviral treatment in HBeAg-positive CHB patients.

## 5. Conclusions

In conclusion, serum AFP is a useful biomarker for identifying significant fibrosis, advanced fibrosis, and cirrhosis in HBeAg-positive CHB patients. As it is relatively low-cost and non-invasive, detecting serum AFP may potentially reduce the need for liver biopsy in HBeAg-positive CHB patients, especially in resource-limited settings.

## Figures and Tables

**Table 1:** General characteristics of the study subjects.

Variables	All Patients, *n* = 276	Normal AFP, *n* = 217	High AFP, *n* = 59	*p*-Value
Age (years)	33.00 (27.00–41.00)	33.00 (25.00–40.00)	34.00 (28.00–43.00)	0.175
Male gender (*n*)	224 (81.2%)	176 (81.1%)	48 (81.4%)	0.965
TP (g/L)	66.90 (63.30–71.70)	66.90 (63.30–71.70)	67.70 (62.70–71.40)	0.936
ALB (g/L)	40.55 (37.63–43.10)	41.00 (38.10–43.65)	39.10 (35.00–42.70)	0.004
GLB (g/L)	26.73 ± 4.74	26.19 ± 4.30	28.71 ± 5.72	0.002
ALT (U/L)	70.50 (52.00–111.75)	65.00 (51.00–101.00)	97.00 (64.00–235.00)	<0.001
AST (U/L)	42.00 (32.00–62.00)	39.00 (30.00–54.50)	65.00 (44.00–126.00)	<0.001
TBIL (μmol/L)	13.50 (10.03–19.08)	12.50 (9.55–17.15)	18.70 (11.80–32.00)	<0.001
PLT (10^9^/L)	169.18 ± 60.14	171.88 ± 58.30	159.22 ± 66.06	0.099
APRI	0.68 (0.44–1.12)	0.62 (0.41–0.92)	1.25 (0.69–2.50)	<0.001
FIB-4	1.00 (0.66–1.71)	0.95 (0.62–1.48)	1.50 (0.97–3.10)	<0.001
Fibrosis stages				
S0	7 (2.5%)	7 (3.2%)	0 (0)	0.352
S1	145 (52.5%)	133 (61.3%)	12 (20.3%)	<0.001
S2	82 (29.7%)	65 (30.0%)	17 (28.8%)	0.865
S3	29 (10.5%)	11 (5.1%)	18 (30.5%)	<0.001
S4	13 (4.8%)	1 (0.4%)	12 (20.4%)	<0.001

AFP: Alpha-fetoprotein, TP: Total protein, ALB: Albumin, GLB: Globulin, ALT: alanine aminotransferase, AST: aspartate aminotransferase, TBIL: total bilirubin, PLT: Platelet, APRI: Aspartate aminotransferase (AST)-to-platelet ratio, FIB-4: Fibrosis-4.

**Table 2:** Univariate and multivariate analysis for variables associated with liver fibrosis.

Variables	Univariate		Multivariate	
	OR (95% CI)	*p*-Value	OR (95% CI)	*p*-Value
Significant liver fibrosis (S2–S4)				
Age (years)	1.038 (1.013–1.063)	0.002	1.027 (1.000–1.056)	0.053
Male gender (%)	0.966 (0.526–1.773)	0.911	---	---
TP (g/L)	0.993 (0.973–1.014)	0.533	---	---
ALB (g/L)	1.007 (0.994–1.019)	0.306	---	---
GLB (g/L)	1.047 (0.995–1.101)	0.080	---	---
ALT (U/L)	1.002 (1.000–1.004)	0.021	0.997 (0.993–1.002)	0.269
AST (U/L)	1.008 (1.003–1.014)	0.003	1.008 (0.997–1.020)	0.168
TBIL (μmol/L)	1.055 (1.027–1.084)	<0.001	1.022 (0.994–1.050)	0.119
PLT (10^9^/L)	0.993 (0.989–0.997)	0.001	0.994 (0.989–0.999)	0.018
AFP (ng/mL)	1.109 (1.056–1.165)	<0.001	1.082 (1.031–1.136)	0.001
Advanced liver fibrosis (S3–S4)				
Age (years)	1.033 (1.002–1.065)	0.035	1.025 (0.990–1.062)	0.160
Male gender (%)	0.538 (0.200–1.443)	0.218	---	---
TP (g/L)	0.982 (0.930–1.036)	0.503	---	---
ALB (g/L)	1.011 (0.998–1.024)	0.088	---	---
GLB (g/L)	1.077 (1.008–1.151)	0.028	1.034 (0.955–1.119)	0.411
ALT (U/L)	1.022 (1.000–1.003)	0.033	1.000 (0.996–1.003)	0.792
AST (U/L)	1.004 (1.001–1.007)	0.011	1.005 (0.999–1.011)	0.130
TBIL (μmol/L)	1.016 (1.001–1.031)	0.033	0.997 (0.967–1.018)	0.769
PLT (10^9^/L)	0.990 (0.984–0.997)	0.003	0.992 (0.985–0.999)	0.025
AFP (ng/mL)	1.023 (1.011–1.036)	<0.001	1.021 (1.007–1.035)	0.002
Cirrhosis (S4)				
Age (years)	0.997 (0.944–1.052)	0.909	---	---
Male gender (%)	0.346 (0.044–2.725)	0.314	---	---
TP (g/L)	0.934 (0.851–1.027)	0.157	---	---
ALB (g/L)	0.825 (0.722–0.942)	0.004	0.851 (0.726–0.997)	0.046
GLB (g/L)	1.050 (0.941–1.171)	0.381	---	---
ALT (U/L)	1.002 (1.000–1.004)	0.016	1.001 (0.997–1.005)	0.696
AST (U/L)	1.004 (1.001–1.007)	0.008	1.004 (0.998–1.010)	0.193
TBIL (μmol/L)	1.014 (0.997–1.032)	0.110	---	---
PLT (10^9^/L)	0.987 (0.976–0.998)	0.023	0.987 (0.974–1.001)	0.062
AFP (ng/mL)	1.017 (1.008–1.026)	<0.001	1.013 (1.003–1.023)	0.010

TP: Total protein, ALB: Albumin, GLB: Globulin, ALT: alanine aminotransferase, AST: aspartate aminotransferase, TBIL: total bilirubin, PLT: Platelet, AFP: Alpha-fetoprotein.

## Data Availability

The data sets used during the current study are available from the corresponding author upon reasonable request.
